# A Robust Multiscale and Multiphasic Structure-Based Modeling Framework for the Intervertebral Disc

**DOI:** 10.3389/fbioe.2021.685799

**Published:** 2021-06-07

**Authors:** Minhao Zhou, Shiyin Lim, Grace D. O’Connell

**Affiliations:** ^1^Berkeley Biomechanics Laboratory, Department of Mechanical Engineering, University of California, Berkeley, Berkeley, CA, United States; ^2^Department of Orthopaedic Surgery, University of California, San Francisco, San Francisco, CA, United States

**Keywords:** finite element modeling, multiscale modeling, multiphasic modeling, structure-based modeling, structure-function relationship, bovine caudal disc, intervertebral disc degeneration

## Abstract

A comprehensive understanding of multiscale and multiphasic intervertebral disc mechanics is crucial for designing advanced tissue engineered structures aiming to recapitulate native tissue behavior. The bovine caudal disc is a commonly used human disc analog due to its availability, large disc height and area, and similarities in biochemical and mechanical properties to the human disc. Because of challenges in directly measuring subtissue-level mechanics, such as *in situ* fiber mechanics, finite element models have been widely employed in spinal biomechanics research. However, many previous models use homogenization theory and describe each model element as a homogenized combination of fibers and the extrafibrillar matrix while ignoring the role of water content or osmotic behavior. Thus, these models are limited in their ability in investigating subtissue-level mechanics and stress-bearing mechanisms through fluid pressure. The objective of this study was to develop and validate a structure-based bovine caudal disc model, and to evaluate multiscale and multiphasic intervertebral disc mechanics under different loading conditions and with degeneration. The structure-based model was developed based on native disc structure, where fibers and matrix in the annulus fibrosus were described as distinct materials occupying separate volumes. Model parameters were directly obtained from experimental studies without calibration. Under the multiscale validation framework, the model was validated across the joint-, tissue-, and subtissue-levels. Our model accurately predicted multiscale disc responses for 15 of 16 cases, emphasizing the accuracy of the model, as well as the effectiveness and robustness of the multiscale structure-based modeling-validation framework. The model also demonstrated the rim as a weak link for disc failure, highlighting the importance of keeping the cartilage endplate intact when evaluating disc failure mechanisms *in vitro*. Importantly, results from this study elucidated important fluid-based load-bearing mechanisms and fiber-matrix interactions that are important for understanding disease progression and regeneration in intervertebral discs. In conclusion, the methods presented in this study can be used in conjunction with experimental work to simultaneously investigate disc joint-, tissue-, and subtissue-level mechanics with degeneration, disease, and injury.

## Introduction

Mechanical dysfunction of the intervertebral disc can lead to reduced mobility and debilitating pain (Adams and Roughley, 2006). Disc prolapse and herniation mostly occur in the posterolateral region, where stresses, strains, and intradiscal pressure in the annulus fibrosus (AF) are higher (Shah et al., 1978; Adams and Hutton, 1985; Steffen et al., 1998; O’Connell et al., 2007b; O’Connell et al., 2011; Wilke et al., 2016; Liu et al., 2017). The posterolateral region has also been linked to increasing bulging and protrusion of the nucleus pulposus under fatigue, with some discs experiencing full herniations (Wilke et al., 2016). Previous researchers have tracked progression of disc failure from bulging to herniation (Adams et al., 2000; Vernon-Roberts et al., 2007), but further investigation is limited due to experimental challenges in directly assessing *in situ* mechanics (e.g., fiber mechanics), which result in large variations in reported *in situ* fiber mechanics data. For example, earlier *in vitro* joint-level studies reported AF fiber strains that varied from ∼0.3 to 20% under axial compression, which may cause contradicting predictions regarding the likelihood of disc failure under physiological conditions (Shah et al., 1978; Stokes, 1987; Heuer et al., 2008a,b, 2012; Wang et al., 2009; Spera et al., 2011). Thus, despite recent advancements in experimental techniques, *in situ* fiber mechanics at the joint level remain poorly understood.

Human intervertebral disc cadaveric tissues are the benchmark for spine biomechanics research, but limited tissue availability and challenges in controlling for important variables, such as sex, age, and level of degeneration, can impact study designs (e.g., sample size) and confound results (Iatridis et al., 2005; Alini et al., 2008; Michalek and Iatridis, 2012; Costi et al., 2020). For these reasons, many researchers have resorted to large animal models, including ovine, porcine, and bovine, to investigate intervertebral disc biomechanics (Alini et al., 2008). Particularly, bovine caudal discs are more accessible than human discs, easier to handle than discs from smaller animals (e.g., rat and mouse discs), and have biochemical and mechanical properties similar to human discs (Demers et al., 2004; Beckstein et al., 2008; Showalter et al., 2012; Bezci et al., 2019). Furthermore, previous work demonstrated the effectiveness of using bovine discs to study the effect of injuries and degeneration by effectively inducing injuries (e.g., needle punctures) and degeneration (e.g., enzyme digestion) in the tissues *in vitro* (Korecki et al., 2008a; Roberts et al., 2008; Michalek and Iatridis, 2012). Despite improvements in availability, accessibility, consistency, and ease of manipulation, experimental limitations still prevent assessment of intradiscal deformations and stress distributions between disc components with injuries or degeneration. Instead, *in vitro* studies primarily assess joint-level bulk mechanics, compositional changes, or biological response (Oshima et al., 1993; Korecki et al., 2008a,b; Roberts et al., 2008; Walter et al., 2011; Michalek and Iatridis, 2012; Bezci et al., 2015, 2020a,b; Bezci and O’Connell, 2018). The growing wealth of data that can be obtained from the bovine caudal discs makes it an ideal animal model to develop a validated and comprehensive computational tool to assess *in situ* mechanics. Additionally, because of lower inter-specimen variability, bovine disc models can be more effectively and reliably validated with experimental data than human disc models.

Finite element models (FEM) have been used to complement experimental studies, providing a powerful tool for predicting hard-to-measure, three-dimensional mechanical and biochemical responses (Zhou et al., 2020c). Since the 1970s, FEMs have advanced the field of spinal biomechanics significantly by providing insights into disc joint-level mechanics and tissue-level stress and strain distributions (Shirazi-Adl et al., 1984; Shirazi-Adl, 1992; Galbusera et al., 2011a,b; Schmidt et al., 2013). However, many joint-level FEMs describe disc components as single-phasic elastic or hyperelastic materials and thus do not account for water content (Kurowski and Kubo, 1986; Kim et al., 1991; Rohlmann et al., 2006; Schmidt et al., 2007b), which is a primary constituent in all biological tissues and plays an important role in the tissue’s load-bearing capability (Ateshian et al., 1994). More recent models have accounted for tissue water content by describing disc components as poroelastic materials, which significantly advanced the field by enabling investigations into the stress-bearing role of the interstitial tissue water content, as well as tissue’s time-dependent behavior (Natarajan et al., 2006; Wilson et al., 2007; Galbusera et al., 2011a,b; Barthelemy et al., 2016; van Rijsbergen et al., 2018; Castro and Alves, 2020). However, these models have limited capability in describing the osmotic response, which has been shown to alter mechanical behavior and change with degeneration (Ishihara et al., 1996; Wognum et al., 2006; Wuertz et al., 2007).

In addition to the limitations in accounting for tissue’s fluid content and osmotic response, most FEMs are developed based on homogenization theory, where every model element includes a homogenized description of tissue subcomponents (e.g., fibers and extrafibrillar matrix) and, thus, does not accurately represent the heterogeneous AF native architecture, where fibers and extrafibrillar matrix are distinct materials that occupy separate volumes. As a result, these models are not capable of directly investigating subtissue-level mechanics (e.g., *in situ* fiber or interfibrillar stress and strain distributions; Yin and Elliott, 2005). To address some of these issues, we previously developed and validated a structure-based FEM of the AF that replicated its native tissue architecture, with fiber bundles modeled as a separate material from the extrafibrillar matrix (Zhou et al., 2020a). In this approach, model parameters directly represented tissue mechanical (e.g., modulus, Poisson’s ratio, etc.) or biochemical properties (e.g., proteoglycan content, referential hydraulic permeability, etc.). To account for tissue water content and osmotic behavior, triphasic mixture theory was employed to describe the swelling capacity of the extrafibrillar matrix (Lai et al., 1991; Ateshian et al., 2004). Our model was able to robustly and accurately predict multilamellar AF mechanics under various loading configurations and testing boundary conditions, including uniaxial tension, biaxial tension, and simple shear (Zhou et al., 2020a). More recently, by incorporating a structure-based fiber engagement analysis, we were also able to apply this model to explain the relationship between specimen geometry and AF tensile mechanics that was originally observed by Adams and Green (1993) and Zhou et al. (2020b).

The objective of this study was to expand our structure-based multiscale modeling-validation approach to study joint-level mechanics of the intervertebral disc under both healthy and degenerated conditions. Degeneration has been shown to alter subtissue-level fiber mechanics, which plays an important role in stress distributions, damage accumulation, and bulk tissue failure (Werbner et al., 2019). Understanding mechanisms of stress distribution within the disc and its subcomponents can help develop robust designs for tissue repair or replacement implants, such as tissue engineered discs. Therefore, we (1) developed and validated a joint-level FEM that was capable of investigating the multiscale and multiphasic structure-function relationship in bovine caudal discs, and (2) used the validated FEM to investigate the effect of loading condition and degeneration on multiscale disc mechanics at joint, tissue, and subtissue scales.

## Materials and Methods

### Model Development

**FIGURE 1:**
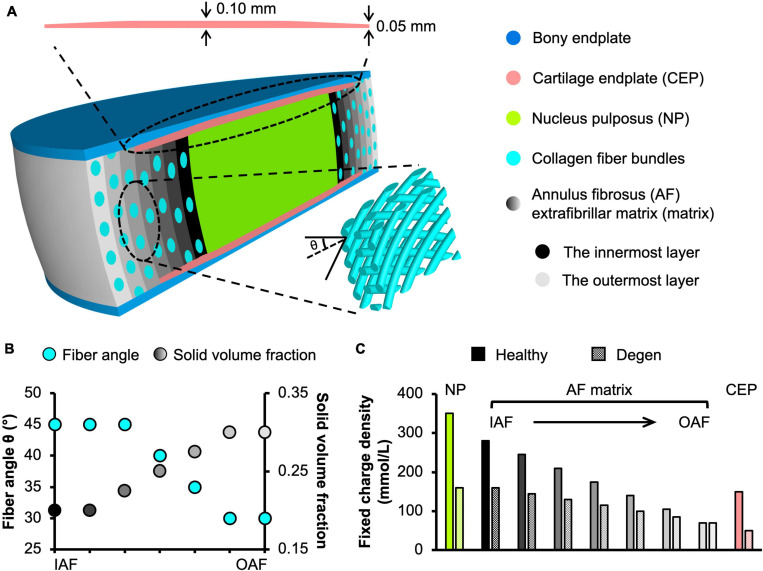
**(A)** Schematic of the multiscale, structure-based bovine caudal disc motion segment model. The extrafibrillar matrix and collagen fibers of the annulus fibrosus (AF) were modeled as distinct materials occupying separate volumes. Insets present the cartilage endplate geometry (top) and the angle-ply fiber structure (bottom right). **(B)** AF fiber angle and solid volume fraction from the inner AF (IAF) to the outer AF (OAF). **(C)** Fixed charge density distribution in healthy and degenerated (Degen) disc models.

FEMs were developed to represent a bone-disc-bone motion segment from the bovine tail (Figure 1A). Neighboring tissues (e.g., facet joints, ligaments, etc.) were not included in the model to minimize confounding effects and to more closely represent motion segment specimens prepared for experimental testing. Model geometry was created in Solidworks (2020) and finite element meshes were generated using ABAQUS and ANSA pre-processor (Abaqus 6.14; ANSA 15.2.0). Mesh size was determined based on results from our previous mesh convergence study (Zhou et al., 2020b). PreView was used to define boundary and loading conditions and the fully developed models were solved by FEBio (PreView 2.1; FEBio 2.8.5; Maas et al., 2012). Due to limited computational resources, the current available solver was only able to process a maximum of ∼200 million non-zero entries in the stiffness matrix. Thus, models created in this study were scaled down at 1:5 scale.

To ensure that this scaling and the resulting changes in the number of AF lamellae modeled did not affect model predictions, preliminary work was performed to determine the effect of scaling ratio between 1:4 and 1:6 on model-predicted compressive and torsional mechanics. Compressive stress-strain behavior and normalized torsional stiffness-rotation response from the 1:4, 1:5, and 1:6 scale models were consistent (Supplementary Figure 1), suggesting that scaling and number of AF lamellae modeled did not affect model predictions when the model included enough AF lamellae. Thus, bovine caudal disc motion segment models were developed at 1:5 scale for computational efficiency (∼2.1 million elements). Finite element meshes of the model were shown in Supplementary Figure 2.

Model geometry was determined based on data reported in the literature. At full scale, the radius and height of bovine caudal discs are 14.20 ± 0.85 mm and 6.90 ± 0.35 mm, respectively, assuming a circular cross section in the transverse plane (O’Connell et al., 2007a). Thus, the 1:5 scaled model radius and height (not including both bony endplates) were created at 2.85 and 1.40 mm, respectively (Figure 1A). The nucleus pulposus (NP) was assumed to have the same circular cross section in the transverse plane, but with a ∼50% smaller radius (1.45 mm; Figure 1A; O’Connell et al., 2007a). The AF was created using our previously reported structure-based modeling approach, where the tissue was described as a fiber-reinforced angle-ply composite containing distinct materials for fiber bundles and the extrafibrillar matrix (Figure 1A; Zhou et al., 2020a). Due to limited computational resources, the native bovine AF structural features, including lamellar thickness, fiber radius, and interfibrillar spacing, were preserved during scaling to reduce the total number of elements needed. This scaling approach, which has been widely applied and validated for human disc models (Shirazi-Adl et al., 1984; Goel et al., 1995a; Galbusera et al., 2011a,b), maintained fiber volume fraction and preserved mesh quality for model convergence and model predictions (Zhou et al., 2020b). As such, seven concentric AF layers were created (lamellar thickness = 0.2 mm; Adam et al., 2015). Fiber bundles were uniformly distributed, full-length cylinders welded to the surrounding matrix (Goel et al., 1995a; Michalek et al., 2009; Schollum et al., 2010). Due to the lack of bovine caudal disc anatomy data in the literature, fiber bundle geometry from the human AF was used, based on the similar collagen networks reported between human and bovine discs (Yu et al., 2002, 2007). Specifically, the fiber bundle radius was 0.06 mm, and interfibrillar spacing within each lamella was 0.22 mm (Marchand and Ahmed, 1990). Fiber angles were oriented at ± 45° to the transverse plane in the inner AF and decreased along the radial direction to ± 30° in the outer AF (Figure 1A–bottom inset; Figure 1B–turquoise circles; Matcher et al., 2004). Cartilage endplates (CEP) covered the superior and inferior ends of the NP and the inner-middle AF (Figure 1A–cartilage endplate); spatial variation in CEP thickness was included based on data in the literature (Figure 1A–top inset; Berg-Johansen et al., 2018). Bony endplates were modeled to cover the superior and inferior ends of the disc (Figure 1A–bony endplate). All interfaces were defined as welded interfaces (Adam et al., 2015).

Triphasic mixture theory was employed to account for tissue water content and osmotic response (Lai et al., 1991; Ateshian et al., 2004). The Holmes-Mow description was employed to model the strain-dependent tissue permeability (*k*) of the NP, AF, and CEP (Eq. 1), where *J* was the determinant of the deformation gradient tensor (*F*), *k*_*0*_ represented hydraulic permeability in the reference configuration, φ_*0*_ represented tissue solid volume fraction, and *M* represented the exponential strain-dependence coefficient. Tissue fluid phase model parameters were determined based on reported values for bovine tissues when available (Table 1–Fluid phase). AF solid volume fraction (i.e., 100% minus water content as a percentage) varied linearly along the radial direction, increasing from 0.2 in the inner AF to 0.3 in the outer AF (Table 1 and Figure 1B–grayscale circles). Fixed charge density represented proteoglycan content in the NP, CEP, and AF extrafibrillar matrix, allowing for osmotic swelling. Radial variation in fixed charge density was determined based on our recent work that provided high-spatial-resolution measurements of bovine caudal disc biochemical composition (Figure 1C–solid bars; Bezci et al., 2019). The collagen fiber bundles were assumed to have no swelling capability (i.e., zero fixed charge density). Free diffusivity (*D*_0_) and within-tissue diffusivity (*D*) of Na^+^ and Cl^–^ were set based on data reported in Gu et al. (2004); 100% ion solubility was assumed (*D*_0,*Na*^+^_ = 0.00116mm^2^/s; *D*_0,*Cl*^−^_ = 0.00161mm^2^/s; *D*_*Na*^+^_ = 0.00044mm^2^/s; *D*_*Cl*^−^_ = 0.00069 mm^2^/s). The solution osmotic coefficient (0.927) was determined based on a linear interpolation of data reported in Robinson and Stokes (1949) and Partanen et al. (2017).

**TABLE 1:** Triphasic material properties of the bovine caudal disc tissues.

NP			AF		CEP
			Matrix	Fibers	
Fluid phase	φ_*0*_	0.2^a^	See Figure 1B^a^		0.4^c,^*
	*k*_*0*_ × 10^–16^ (m^4^/Ns)	5.5^b^	64^b^	64^b^	5.6^c,^*
	*M*	1.92^c,^*	4.8^c,^*	4.8^c,^*	3.79^c,^*
Solid phase	*E* (MPa)	0.4^b^	0.74^b^	0.74^b^	0.31^g^
	ν	0.24^d^	0.16^c,^*	0.16^c,^*	0.18^c,^*
	β	0.95^c,^*	3.3^c,^*	3.3^c,^*	0.29^c,^*
	*E*_*lin*_: (MPa)	N.A.	N.A.	600^e^	N.A.
	γ	N.A.	N.A.	5.95^f,^*	N.A.
	λ_*0*_	N.A.	N.A.	1.05^e^	N.A.

NP, nucleus pulposus; AF, annulus fibrosus; CEP, cartilage endplate; φ_*0*_, solid volume fraction; *k*_*0*_, referential hydraulic permeability; *M*, exponential strain-dependence coefficient for permeability; *E*, Young’s modulus; ν, Poisson’s ratio; β, exponential stiffening coefficient of the Holmes–Mow model; *E*_*lin*_, collagen fiber bundle linear-region modulus; γ, collagen fiber bundle toe-region power-law exponent; λ_*0*_, collagen fiber bundle toe- to linear-region transitional stretch.

* The parameter was determined based on experimental studies using matching human intervertebral disc tissues due to the lack of corresponding data obtained from bovine caudal disc tissues.

*^*a*^Beckstein et al. (2008).*

*^*b*^Périé et al. (2005).*

*^*c*^Cortes et al. (2014).*

*^*d*^Farrell and Riches (2013).*

*^*e*^Fratzl et al. (1998), Gentleman et al. (2003), van der Rijt et al. (2006), and Shen et al. (2008).*

*^*f*^Zhou et al. (2020a).*

*^*g*^Wu et al. (2015).*

(1) $$k\,\left(J\right)=k_{0}\,{\left(\frac{J-\varphi_{0}}{1-\varphi_{0}}\right)}^{2}\,e^{\frac{1}{2}\,M\,\left(J^{2}-1\right)}$$

To describe NP, CEP, and AF extrafibrillar matrix mechanics, a compressible hyperelastic Holmes-Mow material description was used (Eqs 2–4; Cortes et al., 2014). Particularly, *I*_*1*_ and *I*_*2*_ represented the first and second invariants of the right Cauchy-Green deformation tensor, **C**(**C** = **F**^*T*^**F**), *E* represented Young’s modulus, *v* represented Poisson’s ratio, and β represented the exponential stiffening coefficient. AF collagen fibers were modeled using the same compressible hyperelastic Holmes-Mow ground matrix but reinforced with a power-linear fiber description to account for AF non-linearity and anisotropy (Eq. 5). γ represented the power-law exponent in the toe region, *E*_*lin*_ represented the fiber modulus in the fiber linear region, and λ_*0*_ represented the transition stretch between the toe and linear regions (Holzapfel and Ogden, 2017). *B* was a function of γ, *E*_*lin.*_, and λ_*0*_ ($B=\frac{E_{l\,i\,n}}{2}\,\left(\frac{\left(\lambda_{0}^{2}-1\right)}{2\,\left(\gamma -1\right)}+\lambda_{0}^{2}\right)$. Solid phase parameters were determined based on bovine experimental studies when available (Table 1–solid phase), and collagen fiber properties were determined based on type I collagen uniaxial tensile test experimental data (Table 1–solid phase: *E*_*lin*_, γ, and λ_*0*_). For all material properties, data from healthy human discs was used when bovine properties were not available, due to similarities in tissue properties (Table 1–“^∗^”).

(2) $$W\,\left(I_{1},I_{2},J\right)=\frac{1}{2}\,c\,\left(e^{Q}-1\right)$$

(3) $$\begin{array}{l} Q=\frac{\text{β}(1+\text{ν})(1-2\text{ν})}{E(1-\text{ν})}[(\frac{E}{1+\text{ν}}-\frac{E\text{ν}}{\left(1+\text{ν})(1-2\text{ν}\right)})(I_{1}-3)\\ +\frac{E\text{ν}}{\left(1+\text{ν})(1-2\text{ν}\right)}(I_{2}-3)-(\frac{E}{1+\text{ν}}+\frac{E\text{ν}}{\left(1+\text{ν})(1-2\text{ν}\right)})lnJ^{2}] \end{array}$$

(4) $$c=\frac{E\,\left(1-\nu \right)}{2\,\beta \,\left(1+\nu \right)\,\left(1-2\,\nu \right)}$$

(5) λn={0                 λn<1El⁢i⁢n4⁢γ⁢(γ-1)⁢(λ02-1)2-γ⁢(λn-1)γ⁢                  1≤λn≤λ0El⁢i⁢n⁢(λn-λ0)+B⁢(λn2-λ02)+El⁢i⁢n4⁢γ⁢(γ-1)⁢(λ02-1)2-γ⁢(λn-1)γ     λn>λ0

Bony endplates were modeled as a compressible hyperelastic material using the Neo-Hookean description (Eq. 6). *I*_*1*_, *I*_*2*_, *J* were defined as above.*E*_*bonyendplates*_ and ν_bony  endplates_ represented the Young’s modulus (12,000 MPa) and Poisson’s ratio (0.3) of the bony endplates, which were determined based on reported data in the literature (Choi et al., 1990; Goel et al., 1995b; Dreischarf et al., 2014).

(6) $$\begin{array}{l} W_{bon\;yendplates}(I_{1},I_{2},J)\\ =\frac{E_{bon\;yendplates}}{4(1+{\text{ν}}_{bon\;yendplates})}(I_{1}-3)-\frac{E_{bon\;yendplates}}{2(1+{\text{ν}}_{bon\;yendplates})}lnJ\\ +\frac{E_{bon\;yendplates}{\text{ν}}_{bon\;yendplates}}{\left(1+{\text{ν}}_{bon\;yendplates})(1-2{\text{ν}}_{bon\;yendplates}\right)}(lnJ{)}^{2} \end{array}$$

### Multiscale Model Validation

Model robustness and accuracy (i.e., predictive power) were evaluated by simulating a range of loading modalities tested in experiments. All models were simulated using steady-state analyses and the model output were evaluated at equilibrium. Model-predicted properties were compared to experimental measurements at the joint, tissue, and subtissue levels.

#### Joint-Level Validation

At the joint level, resting intradiscal pressure, compressive mechanics, and torsional mechanics were evaluated for the motion segment model described in Section “Model Development.” Resting intradiscal pressure was defined as the average NP pressure after swelling and was compared to *in vivo* and *in vitro* intradiscal pressure data (Urban and McMullin, 1988; Ishihara et al., 1996; Sato et al., 1999; Wilke et al., 1999; Nguyen et al., 2008). Both human intervertebral disc and bovine caudal disc intradiscal pressure data were included for validation, because previous studies have shown similar results between the two species (Oshima et al., 1993; Ishihara et al., 1996; Alini et al., 2008).

Disc compressive and torsional mechanics were evaluated by applying loading protocols described in corresponding experimental studies (Beckstein et al., 2008; Showalter et al., 2012). After swelling (triphasic) in 0.15 M phosphate-buffered saline, compressive mechanics were evaluated by applying a 0.5 MPa axial compression. Boundary conditions at the top and bottom bony endplates were defined to represent boundary conditions reported in Beckstein et al. (2008). The normalized compressive stiffness was calculated as the slope of the model-predicted compressive load-displacement curve in the linear region, which was then normalized by the model geometry (i.e., cross-sectional area and height; Beckstein et al., 2008). Torsional mechanics were evaluated by applying a 0.5 MPa axial compressive preload immediately followed by a 10° axial rotation. Boundary conditions at the top and bottom bony endplates were defined to represent boundary conditions reported in Showalter et al. (2012). Normalized torsional stiffness was calculated by normalizing the slope of the torque-rotation curve between 7.5° and 10° by the model polar moment of inertia (Showalter et al., 2012; Bezci et al., 2018). The model was considered valid for predicting disc intradiscal pressure and stiffness when model-predicted values were within one standard deviation of reported mean values.

To assess the influence of including water content and osmotic response on predicted mechanical behavior, a 1:5 hyperelastic disc model, which is more commonly used in FEMs of the intervertebral disc, was created. In the model, all disc components were modeled using hyperelastic material descriptions, and its compressive stiffness was evaluated by applying a 0.5 MPa axial compression and calculating the slope of the linear region of the stress-strain curve.

#### Tissue-Level Validation

**FIGURE 2:**
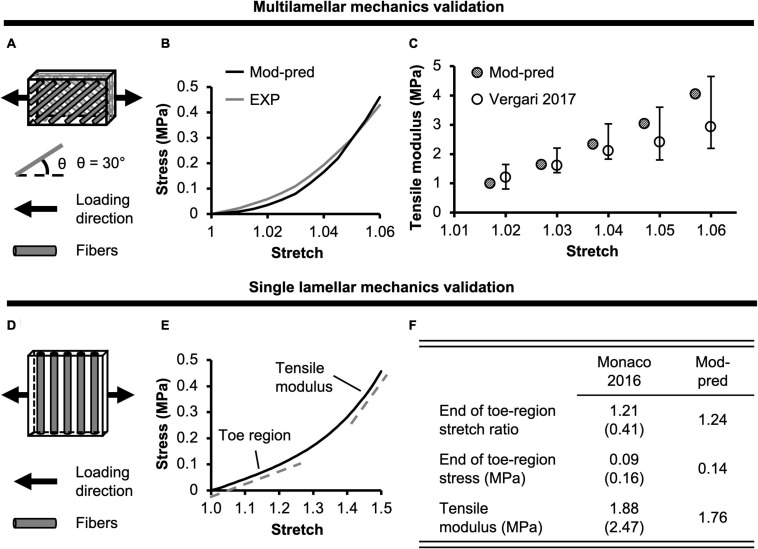
**(A)** Model validation schematic for multilamellar mechanics of bovine annulus fibrosus (AF). Model geometry and loading conditions were determined based on protocols reported in Vergari et al. (2017). **(B)** Model-predicted (Mod-pred) bovine AF multilamellar stress-stretch response compared to representative experimental (EXP) data from Vergari et al. (2017). **(C)** Model-predicted tensile modulus at five specified stretch ratios compared to experimental data from Vergari et al. (2017). **(D)** Model validation for single lamellar mechanics of bovine AF. Model geometry and loading conditions were determined based on protocols reported in Monaco et al. (2016). **(E)** Model-predicted bovine single lamellar stress-stretch response. **(F)** Model-predicted bovine AF single lamellar tensile mechanical properties compared to experimental data [mean (standard deviation)] from Monaco et al. (2016).

At the tissue level, both model-predicted AF mechanical properties and swelling properties were evaluated for model validation. A structure-based FEM was created for bovine multilamellar AF tissue specimens to simulate uniaxial tensile tests performed by Vergari and coworkers (Figure 2A; Vergari et al., 2017). After swelling (triphasic) in 0.15 M phosphate-buffered saline, a 1.1 uniaxial tensile stretch was applied along the circumferential direction (Figure 2A). Boundary conditions were defined to represent no slipping between the grips and the multilamellar tissue sample surface, as reported in Vergari et al. (2017). Tensile modulus was calculated as the slope of the stress-stretch curve at stretch ratios between 1.02 and 1.06 in 0.01 increments, as reported in the literature (Vergari et al., 2017). Tissue explant models of the NP and inner-middle AF were created to evaluate model-predicted swelling behavior in 0.15 M phosphate-buffered saline. Swelling ratios were calculated as the difference between post- and pre-swelling weight divided by the tissue pre-swelling weight and compared to data reported in Bezci et al. (2019). If model-predicted mechanical and swelling properties were within one standard deviation of reported mean values, the model was considered valid for predicting the respective behavior.

#### Subtissue-Level Validation

At the subtissue level, model-predicted AF mechanics were evaluated for model validation. A structure-based model was created for bovine single lamellar AF specimens to simulate uniaxial tensile tests performed by Monaco and coworkers (Figure 2D; Monaco et al., 2016). After swelling (triphasic) in 0.15 M phosphate-buffered saline, a 1.5 uniaxial tensile stretch was applied to the specimen transverse to the fiber direction (Figure 2D). Boundary conditions were defined to effectively replicate the flexible rake system applied in Monaco et al. (2016). Model-predicted uniaxial tensile mechanics were only assessed transverse to the fiber direction, because to the best of the authors’ knowledge, no studies have evaluated bovine single lamellar AF mechanics along the fiber direction analogous to Holzapfel and coworkers’ work using the human AF (Holzapfel et al., 2005). Tensile modulus was calculated as the slope of the stress-stretch curve in the linear region. The model-predicted mechanical properties, including modulus and the stress and strain at the end of the toe-region, were compared to experimental data (Monaco et al., 2016). The model was considered valid for predicting subtissue-level mechanics if the model-predicted mechanical properties were within one standard deviation of reported mean values.

### Effect of Loading Condition on Multiscale Bovine Caudal Disc Mechanics

After validation, three loading conditions were applied to the motion segment model described in Section “Model Development” to evaluate the effect of loading condition on multiscale bovine caudal disc mechanics. All three cases were loaded in two steps. First, swelling in 0.15 M phosphate-buffered saline was simulated. Then, one of the three loading conditions was assessed, including Case *A*: 0.5 MPa axial compression, **Case *B***: 10° axial rotation, and **Case *C***: 0.5 MPa axial precompression followed by 10° axial rotation. For **Case** ***A***, axial compression was simulated between 0–1.0 MPa, but only data from 0.5 MPa axial compression was presented, as it corresponded to experimental data reported in the papers that we compared and validated our model to (Beckstein et al., 2008; Showalter et al., 2012; Bezci et al., 2018). Additionally, the 0.5 MPa axial compression more closely mimicked the compressive stress observed in low-intensity daily activities (e.g., relaxed standing and sitting, walking, etc.; Wilke et al., 1999). For **Cases *B*** and ***C***, disc height was not allowed to change during rotation. Model boundary conditions were defined as in Section “Multiscale Model Validation,” while **Cases *B*** and ***C*** shared identical boundary conditions. All models were simulated using steady-state analyses with the output evaluated at equilibrium. The effect of loading condition was evaluated at the joint, tissue and subtissue levels, as follows:

#### Joint-Level Mechanics

Average solid stress (i.e., stress absorbed by tissue solid matrix) and fluid pressure (i.e., stress absorbed by the tissue interstitial fluid) of the entire bovine caudal disc, including the NP, AF, and CEP, were evaluated for all three cases. The relative contribution of solid stress was evaluated as the solid stress divided by the total stress, which was calculated as the sum of solid stress and fluid pressure based on triphasic mixture theory (Lai et al., 1991). Similarly, the relative contribution of fluid pressure was calculated by normalizing the fluid pressure by the total stress.

#### Tissue-Level Mechanics

NP, AF, and CEP *in situ* swelling ratios were evaluated post-swelling. After the applied mechanical loading, average solid stress, strain, and fluid pressure in the NP, AF, and CEP were evaluated for all three cases. For each disc component, the relative contribution of the solid stress and fluid pressure to the total stress was evaluated. The total stress was calculated as the sum of the component’s solid stress and fluid pressure. Disc bulging of the inner and outer AF was assessed under 0.5 MPa axial compression (**Case *A***) and was calculated by dividing the respective change in mid-disc-height radius with loading by the post-swelling disc radius (reported as a percentage value).

#### Subtissue-Level Mechanics

Average fiber stretch was evaluated within each AF lamellae after swelling and after loading. Swelling-induced fiber stretch was calculated as the post-swelling fiber length divided by the initial fiber length. Post-loading fiber stretch was calculated as the post-mechanical loading fiber length divided by the post-swelling fiber length. Average solid stress in the fibers and extrafibrillar matrix was evaluated post-loading. The relative solid stress contribution of collagen fibers and extrafibrillar matrix to the overall AF solid stress, which was calculated as the sum of fiber and matrix solid stress, was also assessed. Additionally, post-loading fiber solid stress profiles along the fiber length from the inferior to the superior end of the disc were evaluated in both the inner- and outermost AF lamellae.

### Effect of Degeneration on Multiscale Bovine Caudal Disc Mechanics

The effect of degeneration on multiscale disc mechanics was investigated under the three loading conditions evaluated in Section “Effect of Loading Condition on Multiscale Bovine Caudal Disc Mechanics.” Degeneration was achieved by reducing tissue proteoglycan content, which was simulated by reducing the fixed charge density in the NP, AF, and CEP (Adams and Roughley, 2006). Bovines are commonly slaughtered between 18 and 24 months and do not experience spontaneous degeneration within that timespan (Alini et al., 2008). Therefore, fixed charge density distribution for the degenerated disc was determined based on trends observed in degenerated human discs (Figure 1C–checkered bars; Urban and Maroudas, 1979; Beckstein et al., 2008; Bezci et al., 2019), as well as data reported from *ex vivo* degeneration models in relevant bioreactor studies (Castro et al., 2014; Paul et al., 2018). All model-predicted properties discussed and evaluated in Section “Effect of Loading Condition on Multiscale Bovine Caudal Disc Mechanics” were evaluated with degeneration. Additionally, model-predicted resting intradiscal pressure, normalized compressive stiffness, and normalized torsional stiffness were also calculated for the degenerated disc model and compared to available experimental data for a more rigorous model validation (Urban and McMullin, 1988; Sato et al., 1999; Showalter et al., 2012; Bezci et al., 2018). All models were simulated using steady-state analyses with the output evaluated at equilibrium.

## Results

### Multiscale Model Validation

#### Joint-Level Validation

**FIGURE 3:**
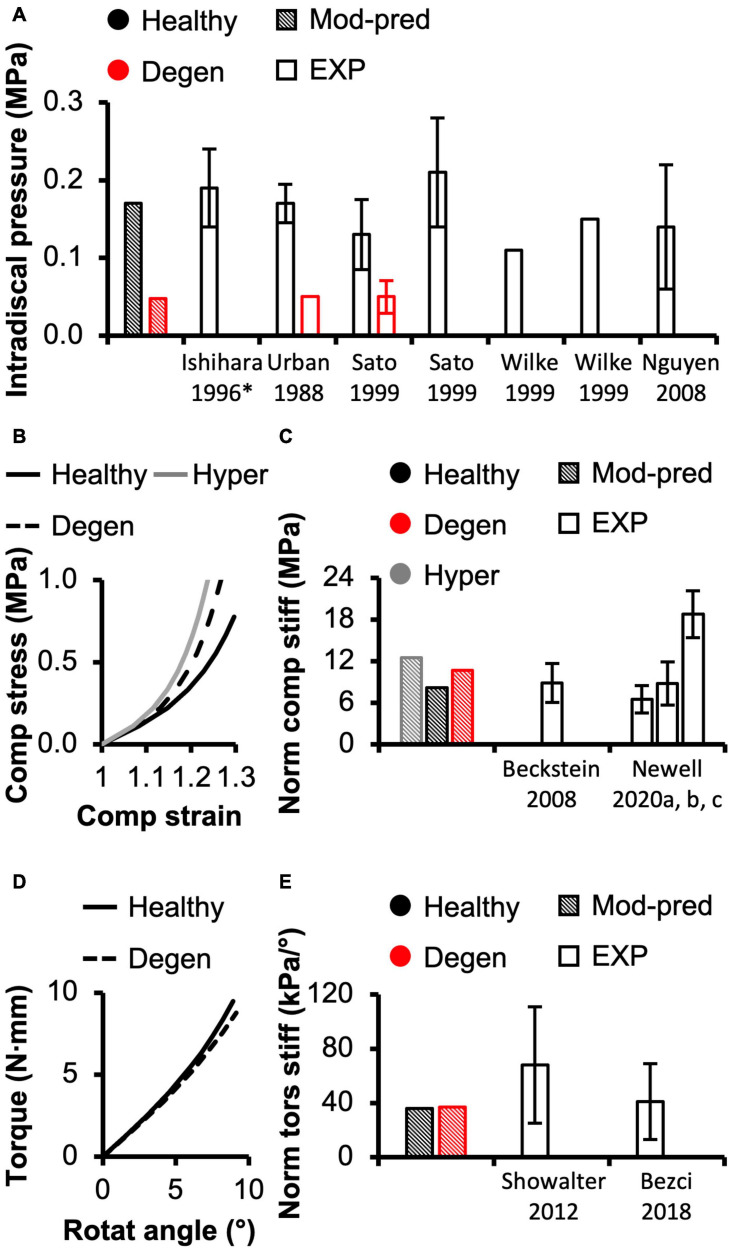
**(A)** Model-predicted (Mod-pred) resting intradiscal pressure in healthy and degenerated (Degen) disc models compared to experimental (EXP) values. Data reported by Ishihara et al. (1996) (noted by *) were obtained from bovine caudal discs while data reported by the other listed studies were obtained from human intervertebral discs, which have shown to share comparable intradiscal pressure values. Variations were not reported in Wilke et al. (1999). **(B)** Representative model-predicted compressive (Comp) stress-strain response of hyperelastic (Hyper), healthy, and degenerated disc models under axial compression. **(C)** Model-predicted normalized (Norm) compressive stiffness (stiff) compared to EXP values. **(D)** Representative model-predicted torsional (tors) response of healthy and degenerated discs when evaluated for torsional mechanics. **(E)** Model-predicted normalized torsional stiffness compared to EXP values.

Model-predicted intradiscal pressure value for the healthy disc was 0.17 MPa, which was within the range of reported experimental values (<0.90× standard deviation from reported mean values; Figure 3A–black diagonal bar vs. white bars enclosed by black lines).

Model-predicted compressive stress-strain response was non-linear for healthy disc models developed using hyperelastic and triphasic mixture theory material descriptions, agreeing well with experimental observations (Figure 3B–solid lines). However, the hyperelastic disc model predicted a stiffer joint-level response than the triphasic model, which accounted for water content and osmotic behavior (Healthy). For the hyperelastic model, predicted normalized compressive stiffness was 12.52 MPa and did not agree with any available datasets (>1.2× standard deviations from reported means). Employing the triphasic material description resulted in a normalized compressive stiffness of 8.12 MPa, agreeing well with Beckstein et al. (2008) and two of three datasets collected, but not published, by Newell et al. (2020) (moduli calculated at a more relevant loading range than the previously published data, see Supplementary Figure 3). Model-predicted compressive stiffness was within 0.8 standard deviation of the reported mean for the three agreed datasets (Figure 3C–black diagonal bar vs. Beckstein et al., 2008 and Newell et al., 2020). However, our model was not able to accurately predict the compressive stiffness reported by the remaining dataset collected for Newell et al. (2020), which represents data from the authors’ own laboratory (18.74 ± 3.35 MPa, Supplementary Figure 3–Berkeley). The model-predicted compressive stiffness was >3.0 × standard deviations from the reported mean of this single dataset since the experimental data from our laboratory was higher than values reported by other institutes (Figure 3C–black diagonal bar vs. Newell et al., 2020).

A pseudo-linear torque-rotation response was observed for the healthy disc (Figure 3D–solid line). Model-predicted normalized torsional stiffness was 36 kPa/°, matching well with reported values (<0.75× standard deviation from the reported mean values; Figure 3E–black diagonal vs. white bars).

#### Tissue- and Subtissue-Level Validation

**FIGURE 4:**
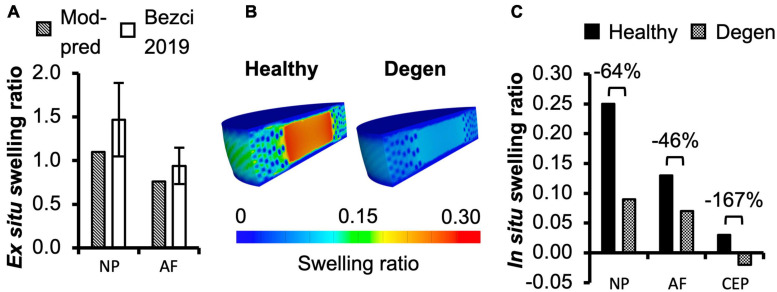
**(A)** Model-predicted (Mod-pred) *ex situ* swelling ratios of the nucleus pulposus (NP) and the inner-middle annulus fibrosus (AF) compared to experimental (EXP) data reported by Bezci et al. (2019). **(B,C)** Model-predicted *in situ* swelling ratios of the NP, AF, and cartilage endplate (CEP) in healthy and degenerated (Degen) disc models. Relative changes in *in situ* swelling ratio with degeneration are labeled above corresponding neighboring bars.

For multilamellar AF specimens, model-predicted stress-stretch response under uniaxial tension was non-linear, agreeing well with the literature (Figure 2B). Model-predicted tensile modulus agreed with the literature but tended to be on the higher end of reported values, particularly as stretch increased (Figure 2C). For single lamellar AF specimens, model-predicted stress-stretch response under uniaxial tension was also non-linear, agreeing well with the literature (Figure 2E). Model-predicted mechanical properties for the toe and linear regions were well within one standard deviation of the reported mean (<0.35× standard deviation from the reported mean; Figure 2F). Based on our model predictions, *ex situ* swelling ratio was 1.10 for the healthy NP tissue and 0.76 for the inner-middle AF, which were both within one standard deviation of the reported means (<0.88× standard deviation; Figure 4A).

### Effect of Loading Condition on Multiscale Bovine Caudal Disc Mechanics

#### Joint-Level Mechanics

**FIGURE 5:**
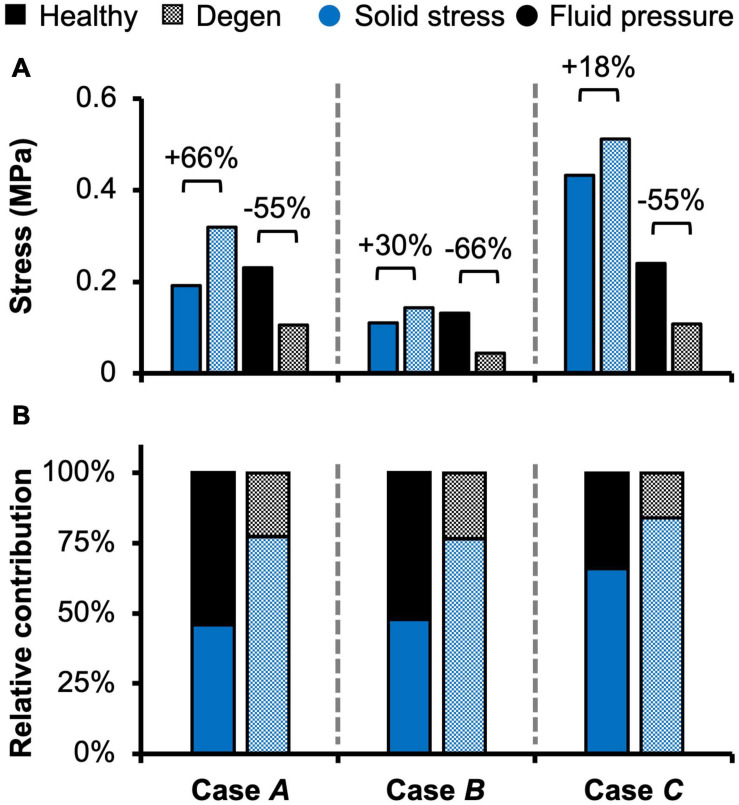
Model-predicted **(A)** solid stress and fluid pressure, as well as **(B)** their relative contribution to the total stress taken by the disc in healthy and degenerated (Degen) models for Cases *A*, *B*, and *C*. Relative changes in solid stress or fluid pressure with degeneration are labeled above corresponding neighboring bars.

Fluid pressure contributed significantly to the disc’s overall load-bearing capacity, especially for loading conditions that incorporated axial compression. In healthy disc models, the average solid stress and average fluid pressure were both approximately 0.2 MPa under axial compression, resulting in relatively equal contribution to the total stress in the disc (Figure 5–**Case *A***). Lower solid stress (0.11 MPa) and fluid pressure (0.13 MPa) were observed under axial rotation, but the relative contribution of solid stress and fluid pressure remained almost identical (Figure 5–**Case *B*** vs. ***A***). Compared to **Case *A***, the combined loading more than doubled the solid stress to 0.43 MPa but did not change the fluid pressure (0.24 MPa). Thus, the resulting relative contribution of the solid stress increased to 64% of the total stress (Figure 5–**Case *C*** vs. ***A***).

#### Tissue-Level Mechanics

**FIGURE 6:**
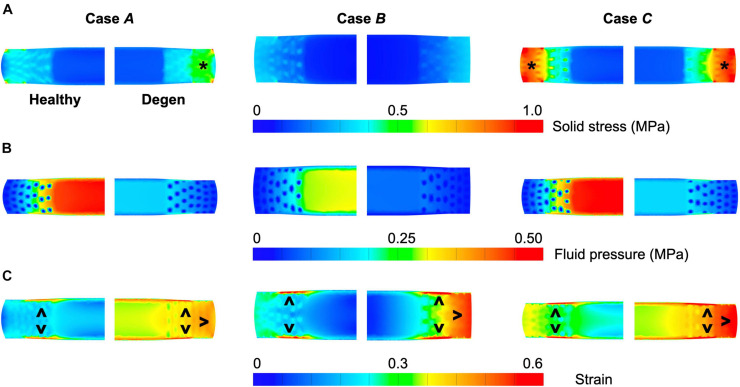
Representative post-loading disc mid-frontal (or coronal) plane **(A)** solid stress, **(B)** fluid pressure, and **(C)** strain distributions in healthy and degenerated (Degen) disc models. Black asterisks highlight stress concentrations. Black triangles point at strain concentrations.

**FIGURE 7:**
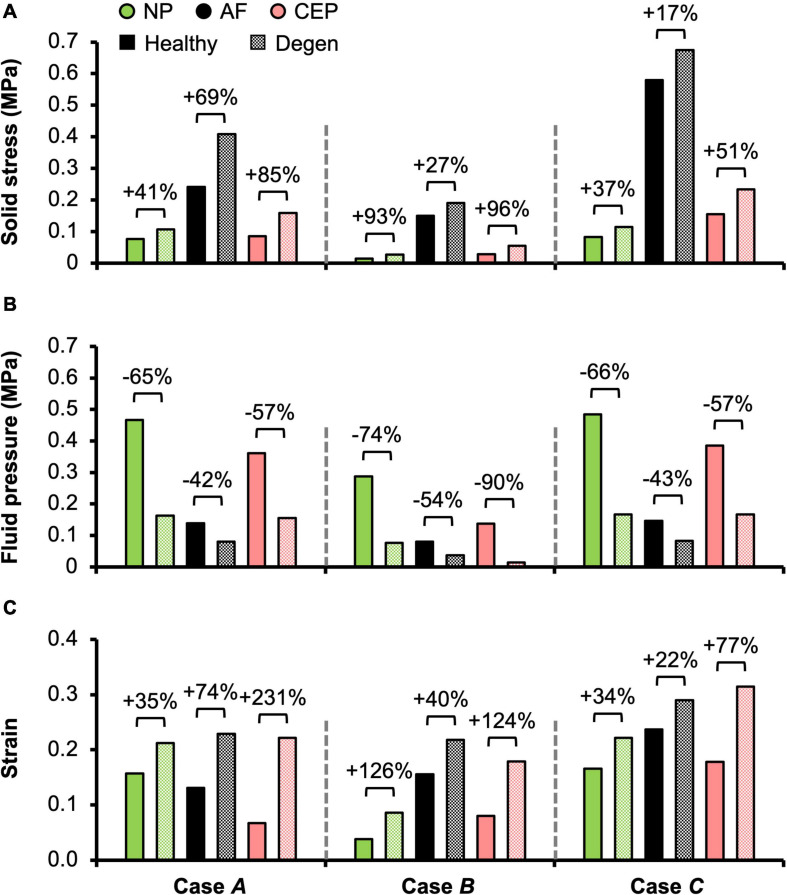
Model-predicted post-loading average **(A)** solid stress, **(B)** fluid pressure, and **(C)** strain in the nucleus pulposus (NP), annulus fibrosus (AF), and cartilage endplate (CEP) in healthy and degenerated (Degen) disc models. Relative changes in NP, AF, and CEP solid stress, strain, or fluid pressure with degeneration are labeled above corresponding neighboring bars.

Different applied boundary and loading conditions resulted in heterogeneous solid stress, fluid pressure, and strain distributions throughout the disc (Figure 6). Large solid stresses were observed in the outer AF, especially in **Cases *A*** and ***C*** (Figure 6A–“**^∗^**”). Compared to **Case *A***, the rotation-only loading condition resulted in lower solid stresses in all disc components (Figure 6A–**Case *B*** vs. ***A***), where the solid stress in the NP, CEP, and AF decreased by more than 80, 67, and 42% (Figure 7A–**Case *B*** vs. ***A***). Under combined loading, a two-fold increase in AF and CEP average solid stress was observed (Figure 7A–**Case *C*** vs. ***A***: black and pink solid bars). However, the addition of rotation to axial compression did not change the NP solid stress (Figure 7A–**Case *C*** vs. ***A***: green solid bar).

*In situ* swelling ratios for the NP, AF, and CEP were 0.25, 0.13, and 0.03, respectively (Figure 4B–Healthy; Figure 4C–black solid bars). Under axial compression, average fluid pressure was 0.14 MPa in the AF, which was ∼70% lower than that in the NP (0.47 MPa) and ∼60% lower than that in the CEP (0.36 MPa; Figure 7B–**Case *A***: solid bars). Fluid pressure under the torsion-only loading was generally lower than that under the compression-only loading. Particularly, compared to **Case *A***, NP and AF fluid pressure were both ∼40% lower while CEP fluid pressure was ∼60% lower (Figure 7B–**Case *B*** vs. ***A***). Interestingly, compared to the compression-only loading condition, combining axial compression with rotation did not have a significant effect on the fluid pressure in any disc components (Figure 7B–**Case *C*** vs. ***A***).

**FIGURE 8:**
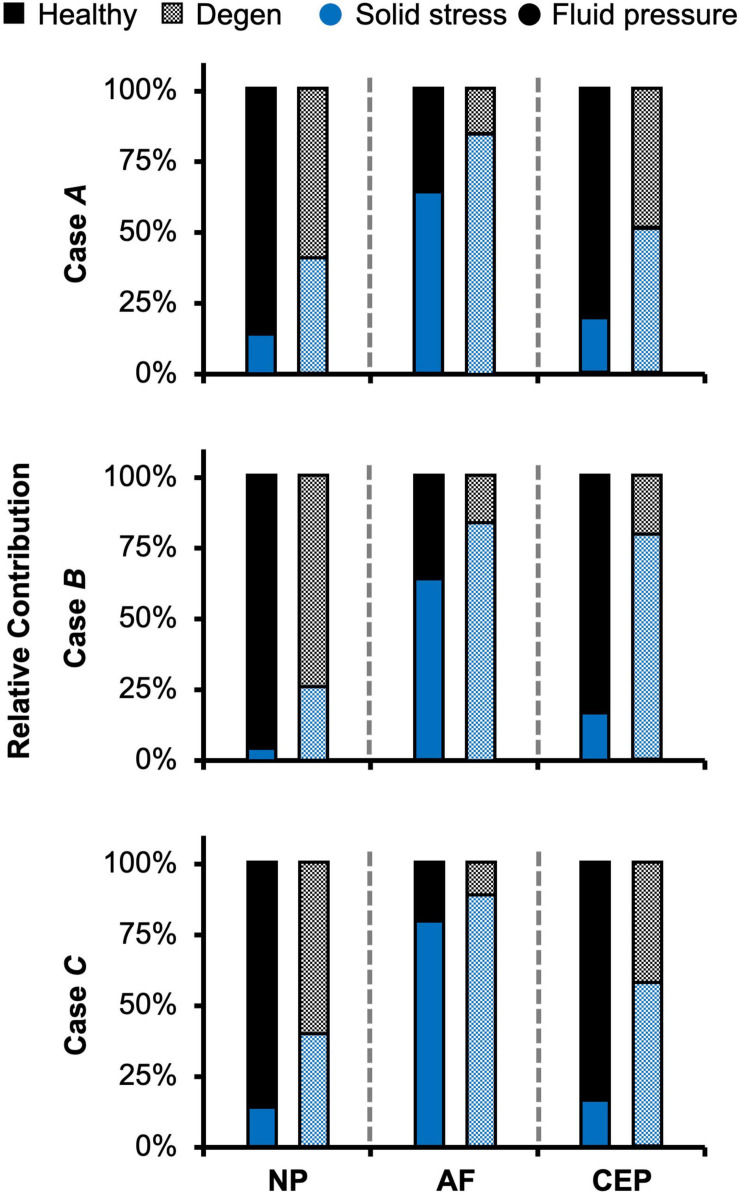
Model-predicted relative contribution of solid stress and fluid pressure in the nucleus pulposus (NP), annulus fibrosus (AF), and cartilage endplate (CEP) in healthy and degenerated (Degen) disc models for Cases *A*, *B*, and *C* after the applied mechanical loading.

As expected, the relative fluid pressure to the total stress was significant and tissue-specific. Across all three loading conditions, fluid pressure accounted for more than 85% of the total stress in the NP and more than 70% of the total stress in the CEP (Figure 8–NP). The relative contribution of fluid pressure was smaller in the AF, but nevertheless accounted for 20–36% of the total AF stress (Figure 8–AF). Compared to the compression-only loading condition, the torsion-only loading resulted in a slight increase in the relative fluid pressure in the NP (Figure 8–**Case *B*** vs. ***A***). However, the combined loading did not alter the relative solid stress or fluid pressure contribution in the NP but resulted in a ∼25% larger solid stress contribution in the AF (Figure 8–**Case *C*** vs. ***A***). The relative solid and fluid contribution in the CEP was not affected by applied loading conditions (Figure 8–CEP).

Large strains were observed at the AF-NP-CEP interface (i.e., the rim) and in the outer AF (Figure 6C–“**^**”). Under axial compression, NP and AF strains were comparable (0.16 and 0.13, respectively) and were approximately twofold greater than strains in the CEP (0.07; Figure 7C–**Case *A***). Under axial rotation, strains in the NP decreased by ∼75%; however, AF and CEP strains increased by ∼20% (Figure 7C–**Case *B*** vs. ***A***). Compression combined with rotation increased AF strains by 80% from 0.13 to 0.24 and increased CEP strains by more than 200% from 0.07 to 0.18. However, the combined loading did not greatly alter NP strains (∼5% change; Figure 7C–**Case *C*** vs. ***A***).

**FIGURE 9:**
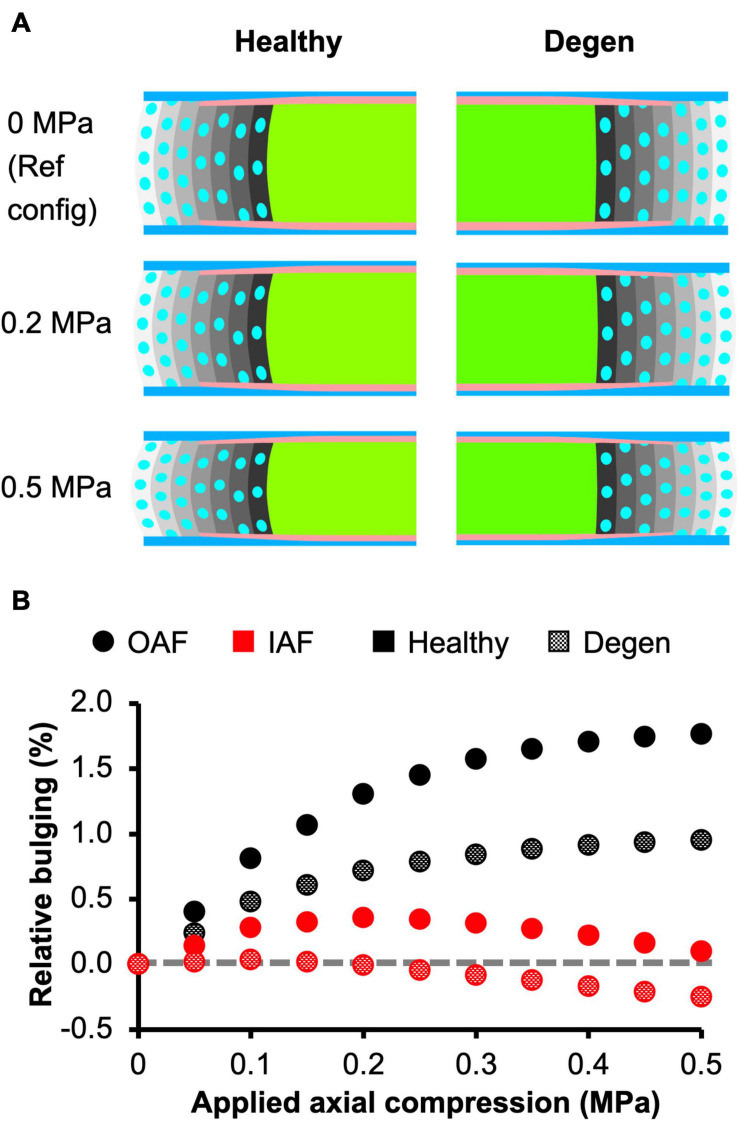
**(A)** Disc mid-frontal (or coronal) cross sections demonstrating the relative annulus fibrosus (AF) bulging in healthy and degenerated (Degen) disc models under axial compression. The relative AF bulging was calculated using the post-swelling 0 MPa configuration as the reference configuration (Ref config). **(B)** Relative bulging in the inner and outer AF in healthy and degenerated disc models. Positive and negative relative bulging suggest outward and inward AF bulging compared to the reference configuration, respectively. The gray horizontal dashed line represents the relative disc bulging threshold, below which the AF was predicted to bulge inward compared to the reference configuration.

Assessment of AF radial displacement at the mid-disc height under axial compression showed outward bulging for both the inner and outer AF after swelling (Figure 9A). In the outer AF, the relative outward bulging increased with applied load, reaching ∼1.8% under 0.5 MPa axial compression (Figure 9B–black solid circles). In the inner AF, the relative bulging reached a maximum of ∼0.4% under 0.2 MPa of compression but then decreased with additional applied compressive load (Figure 9B–red solid circles).

#### Subtissue-Level Mechanics

**FIGURE 10:**
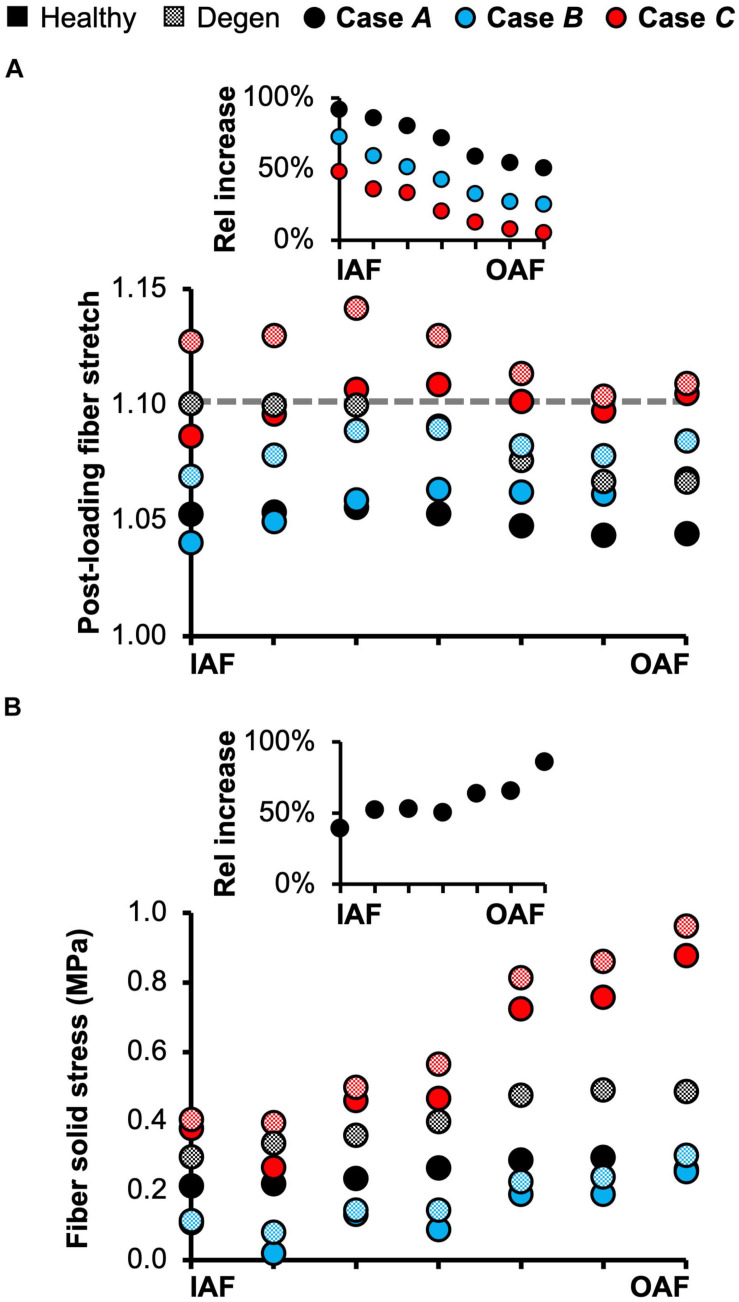
**(A)** Model-predicted average annulus fibrosus (AF) post-loading fiber stretch along the disc radial direction from the inner AF (IAF) to outer AF (OAF) in healthy and degenerated (Degen) disc models. The gray horizontal dashed line highlights the fiber stretch threshold, above which the fibers have a more significant chance of failure based on previous experimental observations. The threshold value was determined based on data reported by Skaggs et al. (1994) and Isaacs et al. (2014). The inset presents the relative (Rel) percentage change in average fiber stretch with degeneration along the disc radial direction. **(B)** Model-predicted post-loading average AF solid stress along the disc radial direction from the IAF to OAF. The inset presents the relative increase in fiber solid stress with degeneration for Case *A*.

The triphasic swelling step applied to all model cases prior to the applied mechanical loading resulted in an average swelling-induced fiber stretch of 1.05 in the inner AF and 1.02 in the outer AF. After applying 0.5 MPa of axial compression, the post-loading fiber stretch was ∼1.05 and was relatively consistent throughout the AF (Figure 10A–black solid circles). The magnitude of fiber stretch under the torsion-only loading was comparable, but there was a linear increase in fiber stretch from the innermost AF layer (1.04) to the outermost layer (1.07; Figure 10A–blue solid circles). Under the combined loading, the fiber stretch was nearly twofold greater than that under the single-axis loading conditions and was ∼1.10 through the AF (Figure 10A–red solid circles).

Average fiber solid stress was relatively consistent throughout the AF under axial compression, ranging from 0.22 MPa in the inner AF to 0.29 MPa in the outer AF (Figure 10B–black solid circles). Under the rotation-only loading, fiber stress in the inner AF was 60% lower than the compression-only condition; however, large changes in fiber solid stress were not observed in the outer AF (Figure 10B–blue vs. black solid circles). Under the combined loading, fiber stress increased linearly from 0.37 MPa in the inner AF to 0.80 MPa in the outer AF. Compared to **Case *A***, the fiber stress was increased by 70% in the inner AF and by 300% in the outer AF (Figure 10B–red vs. black solid circles). The solid stress of AF extrafibrillar matrix, as well as its observed trends with loading condition were both comparable to that of the fibers. Thus, across all three loading conditions, AF collagen fibers and extrafibrillar matrix contributed equally to the overall AF solid stress (Supplementary Figure 4).

**FIGURE 11:**
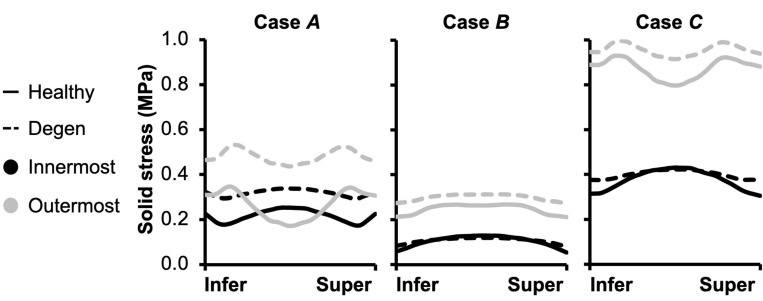
Model-predicted post-loading annulus fibrosus (AF) fiber solid stress profiles along the fiber length from the inferior (Infer) to the superior (Super) bony endplates. The stress distributions were evaluated for the inner- and outermost AF layers in both healthy and degenerated (Degen) discs.

Fiber solid stress profiles were tracked along the fiber length between the inferior and superior bony endplates. In all cases, fiber solid stress distributions were symmetric about the mid-transverse plane, due to disc symmetry (Figure 11). For **Cases *A*** and ***C***, peak fiber solid stresses in the outer AF were observed right below the bony endplates, and peak fiber solid stresses in the inner AF were observed at the mid-disc height (Figure 11–**Cases *A*** and ***C***: solid lines). By contrast, fiber stress was relatively consistent along the fiber length in both the inner and outer AF for **Case *B*** (Figure 11–**Case *B***: solid lines). The combined loading amplified the fiber stress difference between the inner- and outermost lamellae, which shared comparable fiber stresses under the compression- or rotation-only loading conditions (Figure 11–solid black vs. gray lines).

### Effect of Degeneration on Multiscale Bovine Caudal Disc Mechanics

#### Joint-Level Mechanics

Resting intradiscal pressure decreased by ∼70% with degeneration (0.048 MPa) and was within the range of reported values (<0.10× standard deviation from the reported mean values; Figure 3A–red bars). Normalized compressive stiffness increased by ∼30% with degeneration (10.67 MPa; Figures 3B,C). Normalized torsional stiffness was approximately 37 kPa/°, which was not affected by degeneration (Figures 3D,E).

With degeneration, stresses were redistributed with the tissue solid component taking on more of the overall total stress (Figure 5–Degen vs. Healthy). Across the three loading conditions, degeneration increased solid stress by 18–66%, depending on the disc components, and the greatest relative increase with degeneration was observed in the compression-only loading condition (Figure 5A–checkered vs. solid bars). Fluid pressure decreased by ∼60% for all three loading conditions. Thus, the resulting relative contribution of solid stress increased from 45–65% in the healthy discs to 75–85% in the degenerated discs (Figure 5B–checkered vs. solid bars).

#### Tissue-Level Mechanics

As expected, degeneration reduced tissue swelling capability (Figures 4B,C–checkered vs. solid bars). The NP *in situ* swelling ratio reduced by >60%, decreasing from 0.25 to 0.09 with degeneration. Similarly, *in situ* AF swelling ratio decreased by ∼45% from 0.13 to 0.07 with degeneration. Interestingly, the CEP *in situ* swelling ratio became negative (−0.02) in the degenerated disc, indicating a loss of tissue volume after swelling (Figures 4B,C). The decrease in swelling capacity resulted in a 40–90% decrease in fluid pressure, depending on the tissue types and applied loading conditions. Particularly, large degeneration-induced fluid pressure decreases were mostly observed in the NP and CEP (Figure 7B–checkered vs. solids bars).

Similar to joint-level observations, degeneration redistributed stress in each disc component by decreasing the relative contribution of fluid pressure and increasing the relative contribution of solid stress (Figure 8–Degen vs. Healthy). The greatest stress redistribution was observed in the CEP, where the relative fluid pressure contribution decreased from ∼70–80% in the healthy discs to ∼20–50% in the degenerate discs. Noticeably, in **Case *B***, the CEP relative fluid pressure contribution reduced by more than 75% from 83% in the healthy disc to 20% in the degenerate disc (Figure 8–CEP: checkered vs. solid bars). In the NP, the decrease in fluid contribution was relatively consistent for all three loading conditions. Particularly, degeneration reduced NP fluid contribution by ∼20–30%, decreasing from ∼85–95% in the healthy discs to ∼60–75% with degeneration (Figure 8–NP: checkered vs. solid bars). In the AF, the relative fluid pressure contribution decreased by ∼50% with degeneration, ranging from 11 to 17% in the degenerated discs compared to 20–36% in the healthy discs (Figure 8–AF: checkered vs. solid bars). Degeneration also increased the average strain in each disc components by ∼20–240%, with the largest increase observed in the CEP. Similar to the healthy disc, peak strains were observed at the AF-NP-CEP interface (i.e., the rim) and in the outer AF (Figure 6C–“**^**”).

The outer AF was still expected to bulge outward with the level of degeneration simulated in this study. Relative outward bulging for the outer AF at 0.5 MPa axial compression was ∼1%, which was ∼45% smaller than that in the degenerated disc (Figure 9–checkered vs. solid black circles). While the inner AF appeared to bulge outward slightly, calculating the relative change in radial displacement between the post-swelling and post-loading configuration showed that the inner AF moved inward toward the NP by 0.3% (Figure 9A–Degen; Figure 9B–checkered black circles). Although the inner AF moved toward the NP, collapse of the inner AF into the NP, which has been reported for more severely degenerated discs (Adams and Roughley, 2006), was not observed in our model.

#### Subtissue-Level Mechanics

Degeneration increased the average post-loading fiber stretch throughout the AF and had a greater impact on the inner AF than the outer AF (Figure 10A–checkered vs. solid black circles). For **Case *A*,** average fiber stretch decreased linearly from 1.10 in the inner AF to 1.07 in the outer AF (Figure 10A–checkered black circles), representing a 90% increase in fiber stretch in the inner AF and a 50% increase in the outer AF with degeneration (Figure 10A–inset: black circles). For **Case *B***, the average fiber stretch was ∼1.08 and was relatively consistent throughout the AF (Figure 10A–checkered blue circles), where degeneration increased inner AF fiber stretch by more than 70% and increased outer AF fiber stretch by ∼20% (Figure 10A–inset: blue circles). Under the combined loading condition, average fiber stretch exceeded the 1.10 threshold in all AF lamellae, decreasing from 1.14 in the inner AF to 1.11 in the outer AF (Figure 10A–checkered red circles). However, although the inner AF fiber stretch increased by ∼50% with degeneration, the outer AF fiber stretch was not affected (Figure 10A–inset: red circles).

The overall increase in fiber stretch with degeneration did not result in a similar increase in fiber or extrafibrillar matrix solid stress. Under the compression-only loading, solid stress in the fibers increased by more than 40% in the inner AF and by ∼85% in the outer AF (Figure 10B–inset). However, the increases in both fiber and matrix solid stresses were smaller and not as consistent for **Cases *B*** and ***C*** (Figure 10B). Degeneration did not alter the AF fiber/matrix solid stress contribution (Supplementary Figure 4B), nor the pattern of stress distribution along the fiber length, but did increase the stress magnitude, with the largest increase observed for the compression-only loading (Figure 11–dashed vs. solid lines).

## Discussion

This study developed and validated a multiscale and multiphasic structure-based finite element model of the bovine caudal disc motion segment. During development and validation, model parameters were determined based on tissue- or subtissue-level experimental data reported in the literature, as opposed to being calibrated to joint-level mechanics prior to validation. The model validation results highlight the model accuracy and robustness, as well as the advantages of employing the proposed multiscale, structure-based modeling-validation framework. After validation, the model was used to investigate the effect of loading condition and degeneration on solid stress, fluid pressure, and strain distributions at joint, tissue and subtissue scales. While only three loading conditions and one level of degeneration were assessed, results from this study demonstrate the model’s capability in investigating the shifts in disc load bearing or stress distribution mechanisms that can act to induce degenerative remodeling or damage accumulation.

Validation is critical for overall model performance, including accuracy and robustness. Most intervertebral disc models are only validated with respect to global disc measurements, such as axial displacement or intradiscal pressure. This limited validation approach can contribute to inaccurate model predictions, especially at tissue and subtissue scales, where model validation is not usually performed (Shirazi-Adl et al., 1984; Kim et al., 1991; Schmidt et al., 2007b; Galbusera et al., 2011a). Some studies calibrated model parameters, especially those associated with the AF, through optimization algorithms in order for the model predictions to fit experimental datasets measured in tests conducted under specific loading modalities (e.g., axial compression, flexion; Schmidt et al., 2006, 2007a; Malandrino et al., 2013); however, this framework requires models to be recalibrated for each new loading modality or disc geometry. The current study expanded upon our previously reported multiscale validation framework by performing model validation at joint, tissue, and subtissue levels (Zhou et al., 2020a; Figures 3, 4). A total of 16 validation cases were assessed and model-predicted properties agreed well with all but one dataset. Differences in joint stiffness between the outstanding dataset, which originate from our previous work, and our model predictions, are likely caused by the non-ideal machine compliance during experimental data collection (Newell et al., 2020). Importantly, model parameters were directly obtained from tissue- or subtissue-level experimental data and no adjustments were made to match tissue- or joint-level behavior. These results demonstrated the model’s predictive power and the effectiveness of the multiscale validation framework.

The structure-based modeling approach may improve clinical relevance and expand potential use for finite element models of the disc joint. At the tissue level, modeling discrete AF lamellae allowed for reproduction of radial variations in AF biochemical composition (i.e., proteoglycan content and water content). Describing variations in localized proteoglycan content is important for simulating and replicating morphological changes observed with degeneration, including the decrease in disc height, increased outward radial bulging, and inward bulging of the inner AF in severely degenerated discs (Yang and O’Connell, 2019). At the subtissue level, modeling collagen fiber bundles allowed us to explicitly evaluate fiber stress and strain distributions, rather than relying on indirect assessment, such as vector summation to evaluate fiber strain (Schmidt et al., 2007b). The separate fiber bundles generated more realistic predictions of *in situ* fiber mechanics and allowed for direct investigations into fiber-matrix interactions. For example, our findings demonstrate that a ∼50% decrease in proteoglycans caused a 40–90% increase in fiber stress when the disc was loaded under axial compression (Figure 10B–checkered vs. solid black circles). It should be noted that this study only assessed the moderate to severe degeneration level. Thus, additional work is needed to determine whether a decrease in only NP proteoglycan content, as observed in early degeneration, would result in similar increases in fiber stress.

Attributed to the structure-based modeling approach, the majority of our model parameters can be directly linked to tissue mechanical (e.g., modulus, Poisson’s ratio, etc.) or biochemical properties (e.g., water content, proteoglycan content, etc.; Table 1). Model parameters with physical significance help address concerns regarding overparameterization, which is a common issue associated with homogeneous finite element models, where model parameter calibration relies heavily on optimization algorithms (Yin and Elliott, 2005; Eskandari et al., 2019). Taken together, explicitly modeled disc structures with physically relevant model parameters benefit further investigations into disc joint behavior with degeneration, disease, or injury. For example, collagen fiber diameter and stiffness can be readily modified based on structural and mechanical changes noted with degeneration, or diseases such as diabetes (Adams and Roughley, 2006; Li et al., 2013; Svensson et al., 2018). Furthermore, the model can be easily modified to evaluate advanced tissue engineering designs (e.g., angle-ply disc replacements) before conducting costly and time-intensive *in vivo* studies in large animal models (Martin et al., 2014), or to help track time-dependent changes during bioreactor organ cultures (Frauchiger et al., 2018; Pfannkuche et al., 2020).

The importance of accounting for tissue water content and osmotic response was elucidated by assessing the relative stress contribution from tissue solid matrix and interstitial fluid (Figures 5–8). The contribution of fluid pressure plays a pivotal role in the disc’s load-bearing capacity (Adams and Roughley, 2006), but to the best of the authors’ knowledge, it has not been quantified. Inclusion of triphasic material properties allows for direct measurements of fluid pressure. Based on our model predictions for healthy discs, fluid pressure accounted for 35–55% of the total stress (Figure 5). More specifically, the fluid pressure contribution in the NP was greater than 85% (Figure 8), agreeing with previous findings for the healthy articular cartilage, which has a comparable fixed charge density and water content as healthy NP tissues (Maroudas et al., 1969; Armstrong and Mow, 1982; Lüssea et al., 2000; Shapiro et al., 2002). Degeneration reduced tissue swelling capacity, altering the disc’s load-bearing mechanism by shifting more stress to the tissue solid matrix (Figures 5, 8). This shift in stress-bearing was particularly noticeable under axial compression, where the decrease in fluid pressure (i.e., 0.13 MPa) was balanced by an equivalent increase in solid stress (Figure 5A–**Case *A***). Despite the decrease in relative fluid pressure contribution with degeneration, fluid pressure still accounted for up to 25% of the total stress and contributed to more than 60% of NP stress (Figures 5, 8–checkered bars).

Models that do not incorporate tissue swelling describe stress as being entirely absorbed by the solid matrix (single-phasic hyperelastic material description), which likely contributed to overestimations in AF fiber stretch. For example, a previous model that employed single-phasic material descriptions for the disc predicted a fiber stretch of ∼1.12 under the rotation-only loading, even with the inclusion of posterior functional spinal structures (Schmidt et al., 2007b). However, experimental data on AF single lamellar tensile mechanics reported AF fiber bundle failure stretch as 1.14 ± 0.04 (Skaggs et al., 1994; Isaacs et al., 2014). Thus, such a model would suggest a relatively high likelihood of disc failure, contradicting to *in vitro* studies that showed low risk of disc failure under axial rotation (Berger-Roscher et al., 2017). The single-phasic material description may also help explain the overestimated compressive stiffness predicted by our hyperelastic model, as omission of water content and osmotic response led to higher AF solid matrix stress and larger fiber deformations that stiffened the disc joint (Figures 3B,C). Thus, our proposed model can potentially provide valuable insights into cell mechanobiology studies, as more accurate predictions of solid matrix stress and stretch data are required in order to apply physiological loading to cells or tissues *in vitro* (Martin et al., 2014).

The predictive power of our model was further demonstrated by evaluating the multiscale disc mechanics under different loading conditions and degeneration. Single-axis loading conditions (i.e., compression-only or rotation-only) resulted in a fiber solid stress <0.3 MPa and fiber stretch between 1.03 and 1.07 for the healthy disc model, which was comparable to *in situ* subfailure fiber stretch data obtained from photogrammetry-based studies (1.07–1.11; Heuer et al., 2008a,b; Heuer et al., 2012). Taken together, our model predictions for fiber stretch and stress suggest low risks of failure under the single-axis loading conditions, especially under axial rotation, as the average AF fiber stretch did not exceed 1.10 even with degeneration, which agrees well with recent six-degree of freedom testing results (Berger-Roscher et al., 2017). In contrast, multi-axis loading increased the likelihood of damage accumulation and disc failure as axial rotation combined with compression increased the average fiber stretch to 1.10 and almost tripled the average fiber stress in the outer AF from 0.3 to 0.9 MPa, which is much closer to the 1.0 MPa threshold reported in the literature (Skaggs et al., 1994; Holzapfel et al., 2005; Isaacs et al., 2014).

Degeneration increased the fiber stretch and fiber solid stress under all three simulated loading conditions, especially under the compression-only loading (Figure 10–**Case *A*** insets and Figure 11). Interestingly, under the combined loading, the average AF fiber stretch exceeded the 1.10 threshold for failure or significant damage accumulation (range: 1.11–1.14) but the average fiber solid stress still remained below 1.0 MPa. Taken together, these findings suggest that disc failures, especially those initiated in the AF (e.g., clefts, tears, etc.) may be strain-driven rather than stress-driven, agreeing with our previous tissue-level study (Werbner et al., 2017). Six degree of freedom testing machines provide the best approach for elucidating disc failure mechanisms *in vitro* (Costi et al., 2020). However, their high cost and complexity have limited their use. This model may provide a high-throughput approach to better understand the role of complex loading on damage accumulation and ultimate tissue failure (e.g., disc herniation).

Disc failure, especially those induced *in vitro*, have been commonly shown to occur through endplate fracture or annulus prolapse (Adams and Hutton, 1985; Wilke et al., 2016; Berger-Roscher et al., 2017). Across the three loading conditions evaluated, strain concentrations and peak fiber stresses were observed near the NP-AF-CEP interface and at the outer AF, especially in the degenerated disc (Figure 7C–“**^**”; Figure 11–gray solid lines). With degeneration, the CEP exhibited a volume loss post-swelling, likely caused by the compression from surrounding tissues due to differences in swelling capacities (Figure 4C). These results further highlight the NP-AF-CEP interface (i.e., the rim) as a weak link for disc failure. It should be noted that the flatter interface modeled between the CEP and the NP/AF was more representative of discs found in ovine, porcine, and human rather than bovine, which has a more concave CEP-NP-AF interface. Thus, it is within our expectations that our model-predicted peak stress and strain locations match well with *in vitro* failure locations observed in human and ovine discs (Adams and Hutton, 1985; Wilke et al., 2016; Berger-Roscher et al., 2017).

Although this study presents a strong validation and a robust modeling-validation framework, it is not without limitations. First, disc degeneration was simulated by only reducing tissue fixed charge density (i.e., proteoglycan content), without including any degeneration-related structural changes, such as AF lesions and decreased disc height. The omission of these structural or morphological changes might explain model predictions that contradicted previous experimental observations. For example, it has been widely accepted that degeneration results in higher disc flexibility in axial rotation, which was not predicted by our model within the simulated axial rotation range (Mimura et al., 1994; Galbusera et al., 2014). Additionally, previous experimental studies showed that annular bulging increases with degeneration and injury (Heuer et al., 2008b; Zou et al., 2009). While our model accurately predicted relative AF bulging in healthy discs (O’Connell et al., 2007b), it predicted that AF bulging decreased with degeneration (Figure 9–Degen vs. Healthy). Secondly, flexion/extension and lateral bending, which are important physiological loading modalities that have been shown to initiate disc failure at the CEP, were not assessed (Berger-Roscher et al., 2017). Ongoing and future work will include applying this multiscale, structure-based modeling-validation framework to human intervertebral discs to evaluate the risk of disc failure with early to moderate, or even more severe degenerative changes in tissue composition.

This study used a multiscale, structure-based modeling-validation framework to examine multiscale bovine caudal disc mechanics, including but not limited to fluid pressure, solid stress, and fiber stretch and strain. The model accurately predicted variations in disc mechanics under various loading conditions and with degeneration. Importantly, results from this study elucidated important load-bearing mechanisms and fiber-matrix interactions that are important for understanding disease progression and regeneration in intervertebral discs. In conclusion, the methods presented in this study can be used in conjunction with experimental work to simultaneously investigate disc joint-, tissue-, and subtissue-level mechanics with degeneration, disease, and injury.

## Data Availability Statement

The raw data supporting the conclusions of this article will be made available by the authors, without undue reservation.

## Author Contributions

MZ: conceptualization, methodology, software, validation, investigation, data collection and analysis, writing, review and editing, visualization, and project administration. SL: validation, investigation, writing, review and editing, and visualization. GDO: supervision, writing, review and editing, project administration, and funding acquisition. All authors contributed to the article and approved the submitted version.

## Conflict of Interest

The authors declare that the research was conducted in the absence of any commercial or financial relationships that could be construed as a potential conflict of interest.
