# Supplementary material for: A Robust Multiscale and Multiphasic Structure-Based Modeling Framework for the Intervertebral Disc
Source: Front Bioeng Biotechnol. 2021 Jun 7;9:685799. doi: 10.3389/fbioe.2021.685799 (PMC8215504; doi:10.3389/fbioe.2021.685799)
Supplement: Supplementary file 1 [file Data_Sheet_1.docx]

Supplementary Material


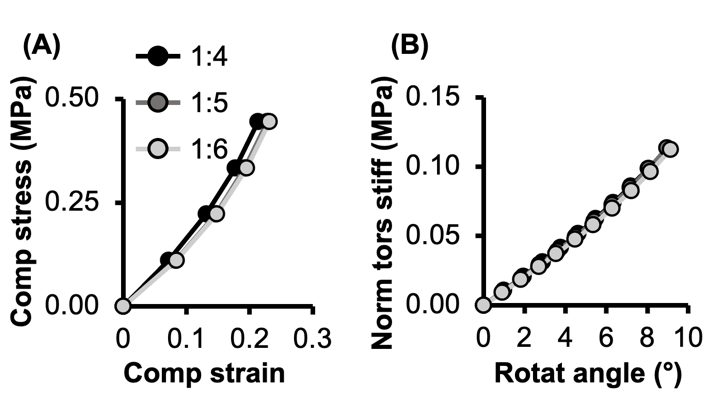


**Supplementary Figure 1.** Model-predicted **(A)** compressive (comp) stress-strain response under axial compression and **(B)** normalized (norm) torsional (tors) stiffness (stiff)-rotation (rotat) response of the 1:4-, 1:5-, and 1:6-scale healthy bovine caudal disc models. The 1:4-, 1:5-, and 1:6-scale models include eight, seven, and six annulus fibrosus lamellae, respectively.


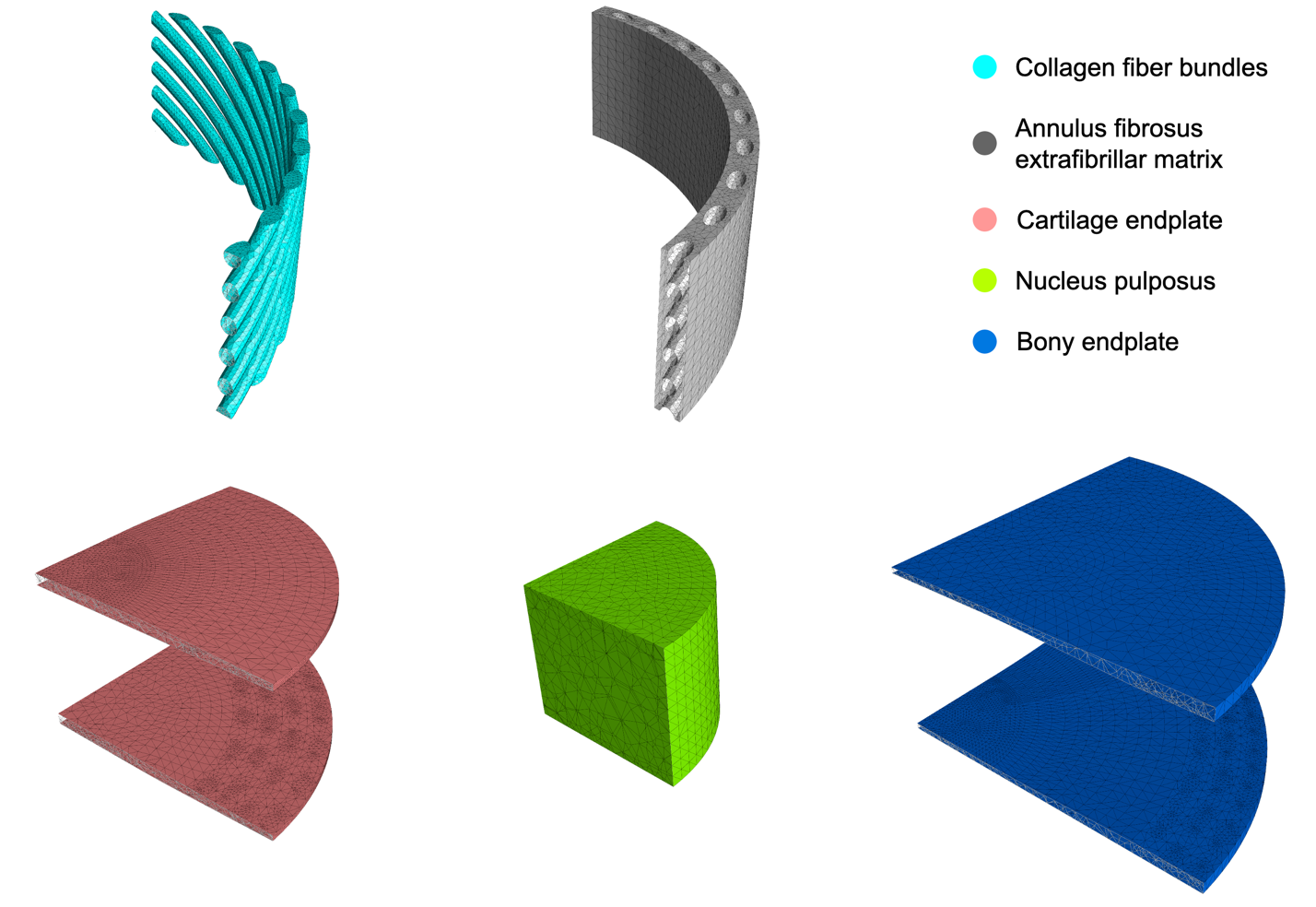
**Supplementary Figure 2.** Finite element meshes of individual disc subcomponents.


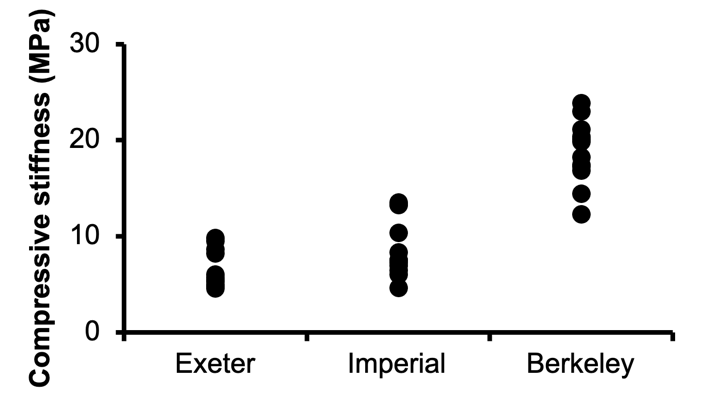


**Supplementary Figure 3.** Experimental bovine caudal disc compressive stiffness calculated at 0.3-0.6 MPa axial compression at three different institutions. The three institutions included University of Exeter (Exeter), Imperial College London (Imperial), and University of California, Berkeley (Berkeley). Data was collected during the study outlined in Newell *et al.* 2020, under the same parameters, and with compressive stiffness calculated between 100N and 300N.


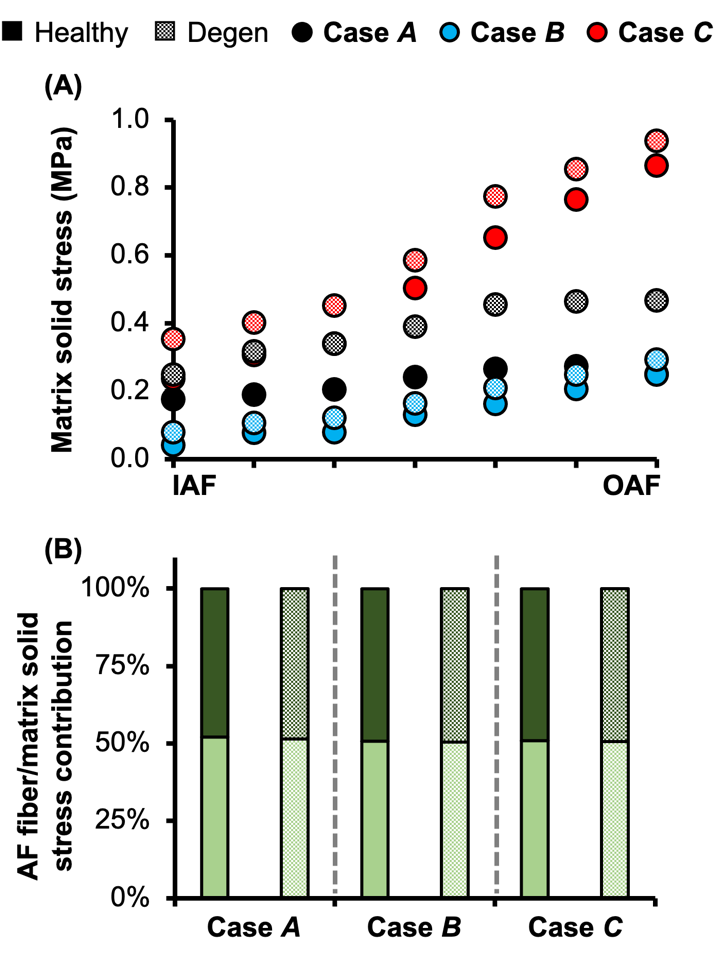


**Supplementary Figure 4. (A)** Model-predicted post-loading average annulus fibrosus (AF) extrafibrillar matrix solid stress along the disc radial direction from the IAF to OAF. **(B)** AF fiber and matrix solid stress contributions in all three cases.
